# Design and Analysis of Electromagnetic-Piezoelectric Hybrid Driven Three-Degree-of-Freedom Motor

**DOI:** 10.3390/s20061621

**Published:** 2020-03-14

**Authors:** Zheng Li, Peng Guo, Zhe Wang, Liang Zhao, Qunjing Wang

**Affiliations:** 1 School of Electrical Engineering, Hebei University of Science and Technology, Shijiazhuang 050018, China; guophb@stu.hebust.edu.cn (P.G.); wangzhe@stu.hebust.edu.cn (Z.W.); zhaoliang@stu.hebust.edu.cn (L.Z.); 2 National Engineering Laboratory of Energy-Saving Motor & Control Technique, Anhui University, Hefei 230601, China; wangqunjing@ahu.edu.cn

**Keywords:** three-degrees-of-freedom, hybrid drive motor, electromagnetic-piezoelectric drive, characteristics analysis

## Abstract

Multi-DOF movement actuators are widely used in industry, mainly in the fields of bionics and precision machining. With the non-stop improvement of modern-day industry, the requirements for the precision, integration and flexibility of multi-degree-of-freedom motion actuators in the industrial field have progressively increased. This paper presents a novel electromagnetic–piezoelectric hybrid driven three-degree-of-freedom motor. The driving method of the hybrid drive motor can be divided into electromagnetic driving and piezoelectric driving. The motor structure and working principle are analyzed. The structural parameters are obtained by modal analysis of the stators and rotor. The rationality of the stator structure is proved by using the transient analysis of the piezoelectric stators. The magnetic field characteristics of the motor are analyzed by both analytical method and the finite element method. The contact pressure and displacement between the piezoelectric stator and the rotor are analyzed by the analytical method. A motor drive model is established, which provides the basis for motor optimization design and control. Finally, a motor prototype and its test platform were built, and the experimental results are presented to verify the rationality of the motor design.

## 1. Introduction

A multi-degree-of-freedom (DOF) motor is a device whose rotor can achieve two degrees of freedom or more degrees of freedom of motion. Multi-DOF motors are widely used in bionic fields and multi-degree-of-freedom motion fields. With the further development of modern intelligent industry, the demand for high-precision, high-integration and high-flexibility multi-DOF motors is rapidly growing. In general, a multi-DOF actuator consists of a few traditional motors [1]. However, the mechanism has a complicated structure, a large volume and low positioning control precision, which cannot meet the requirements of the micro-intelligent drive system [2]. Therefore, the multi-DOF integrated actuator drive technology has attracted the attention of experts and scholars.

In the past decade, many multi-DOF motion devices were developed, which have been widely used in bionic robots and multi-DOF motion platforms [3,4,5,6,7]. Mashimo et al. proposed a spherical ultrasonic motor consisting of three annular stators and a spherical rotor. This design enables three-DOF motion, and simplifies the structure of multi-DOF motors [8]. Guo et al. proposed a small three-DOF ultrasonic motor. It is driven by the first longitudinal and second bending vibration modes. The motor performance was improved through a simple piezoelectric lead zirconatetitanate (PZT)-tube stator [9]. 

Wang, et al., proposed a novel two-DOF spherical ultrasonic motor with three traveling-wave type annular stators to improve the tolerance and controllability of multi-free ultrasonic motors [10]. Chen et al. proposed a novel two-DOF planar linear ultrasonic motor that can achieve the 2-DOF drive function through a variable-mode excitation method [11]. Shi et al. designed a new multi-DOF ultrasonic motor with a ring-shaped composite stator. Large output torque and compact structure are obtained through two orthogonal axial bending modes and a radial bending mode [12]. Liu et al. proposed a sandwich multi-DOF ultrasonic motor with hybrid excitation. The motor is driven through a combination of three resonant modes [13]. The piezoelectric multi-DOF motor motors have the advantages of compact structure, flexible design, easy realization of miniaturization, self-locking, fast response speed, and no external electromagnetic interference [14], so it is suitable for low-speed and high-torque applications. However, it has a short operation life, and it is not suitable for continuous operation. Therefore, Bai et al. proposed a motion platform based on a spherical motor for high-performance nozzle-substrate negotiation in conformal printing of curved electrons. The ball joint type ball motor provides continuous three-DOF rotation, which greatly improves printing efficiency [15]. Li et al. proposed a Halbach array permanent magnet (PM) spherical motor consisting of a spherical rotor and a spherical shell stator. The Halbach array PM structure is used to improve the motor magnetic field distribution and the performance [16]. Lee et al. introduced a spherical multi-DOF motor with double air gaps and proposed a method to improve the motor output characteristics and stability [17]. Li et al. proposed a synthesis strategy for the stator magnetic field of a PM spherical motor to support the further control of the motor. The electromagnetically-driven multi-DOF motor has the characteristics of high mechanical integration and high speed, which is suitable for continuous operation [18]. However, the structure of the electromagnetically-driven multi-DOF motor is relatively complicated, and its magnetic field is difficult to control [19].

The development of hybrid drive multi-DOF motors has gradually become a research trend [20]. The hybrid driven multi-DOF motor mainly combines various drive modes, and hence it does not have the problems of the conventional one drive mode. For example, electromagnetic hybrid-driven multi-DOF motors have problems, such as their difficulty to control the magnetic field, low positioning accuracy and difficulty to transmit high torque through output shaft [21,22], and the multi-DOF motor with piezoelectric hybrid driven has the problems of low operating life and unstable performance [23]. In order to compensate the weaknesses of each type, a novel electromagnetic-piezoelectric hybrid-driven three-DOF is proposed. This hybrid-driven three-DOF motor combines two different driving types. The coarse movement of the motor over a large range is driven by electromagnetic drive. The small movement of the motor is driven by the piezoelectric drive. The electromagnetic-piezo-hybrid driving method not only improves the accuracy of the multi-degree-of-freedom motor, but also realizes the continuous and stable operation of the motor.

In this paper, the basic structure, working principle and hybrid drive strategy of the motor are described. Then, the motor structural model is established by the finite element analysis, and the modal analysis, harmonic response analysis and transient analysis of the motor are carried out. The air gap magnetic field characteristics of the motor are analyzed by analytical method, and the rationality of the results is verified by the finite element method. The contact pressure and contact displacement between the piezoelectric stator and the rotor are analyzed through the analytical method, and the motor drive model is established. Finally, a motor prototype and its motor test platform are built. The design rationality of the motor is proven by the experimental results on the prototype.

## 2. Structure and Principle of the Motor

**Figure 1:**
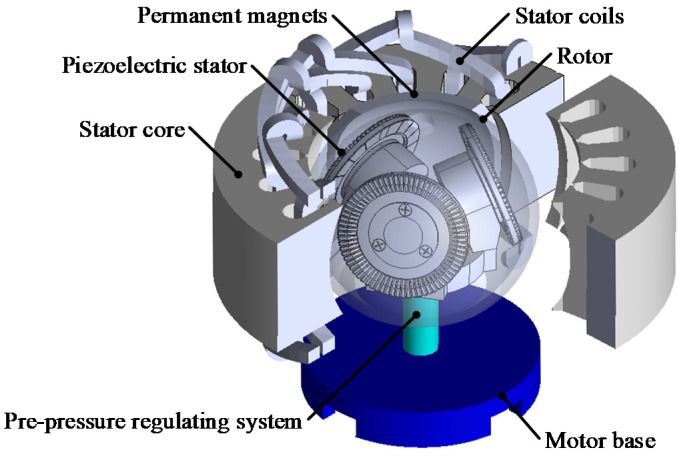
Structure of electromagnetic-piezoelectric hybrid drive motor.

As shown in Figure 1, the electromagnetic-piezoelectric hybrid drive three-DOF motor is mainly composed of the motor base, pre-pressure regulating system, PMs, electromagnetic stator core, electromagnetic stator coils, piezoelectric stators (No.1 stator, No.2 stator and No.3 stator) and rotor. The number of permanent magnets is four, two of which are N poles and two are S poles, which are alternately arranged. 

The permanent magnet is a spherical structure, which is attached to the rotor. The angle (α) between the central axis of the three piezoelectric stators and the horizontal plane is 17.596°. The included angle of the central axis of each stator is 120°. There are four spherical permanent magnets attached to the rotor. The pre-pressure between the stator and the rotor is adjusted by a pre-pressure regulating system.

**Figure 2:**
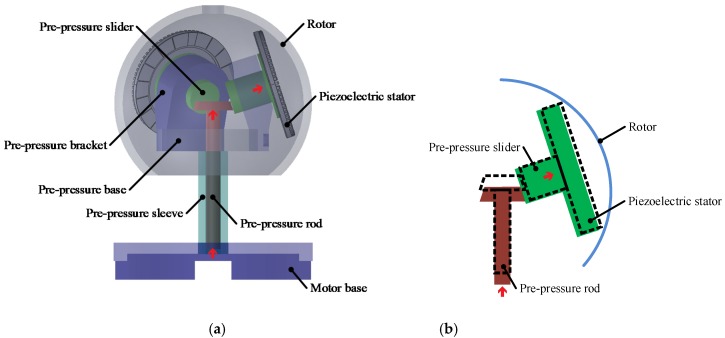
Pre-pressure regulating system: (**a**) Schematic diagram of pre-pressure system (**b**) Pre-pressure regulating diagram.

As shown in Figure 2a, the pre-pressure regulating system is mainly composed of a pre-pressure base, a pre-pressure bracket, a pre-pressure rod, a pre-pressure sleeve and a pre-pressure slider. The pre-pressure sleeve and the motor base are connected by a screw, and the bottom of the pre-pressure rod and the pre-pressure sleeve are connected by another screw. In Figure 2b, the force generated by rotating the pre-pressure lever is converted into the pressure of the piezoelectric stator on the rotor spherical shell. When the pre-pressure bar moves upward, the pre-pressure slider moves in a direction close to the rotor case. In this way, the pre-pressure between the piezoelectric stators and rotor is applied.

**Figure 3:**
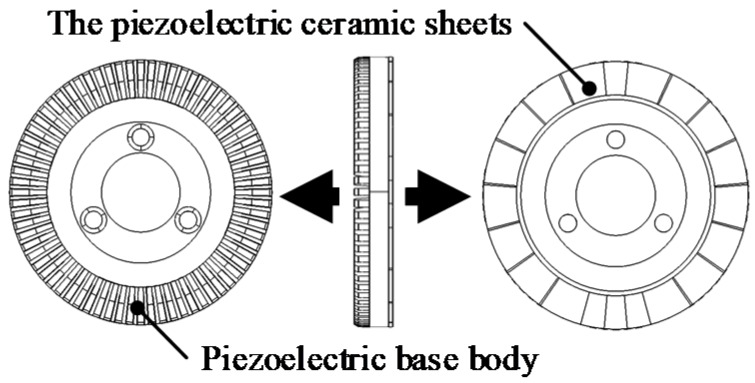
Piezoelectric stator.

As shown in Figure 3, the piezoelectric stator is composed of the piezoelectric base body and the piezoelectric ceramic sheets. Alternating voltages with the same frequency and amplitude, but a phase difference of 90° is applied to the two phases of the piezoelectric ceramic sheets. 

The piezoelectric stator responds to a pulsating wave with a phase difference of 90° in both time and space, which is in accordance with the principle of a piezoelectric inverse effect. A circularly traveling wave is formed by the superposition of the two-phase standing waves. Then the rotor rotates along the tangential direction of the particle surface of the piezoelectric stator surface, and the direction of rotor rotation is opposite to the direction of the traveling wave.

**Figure 4:**
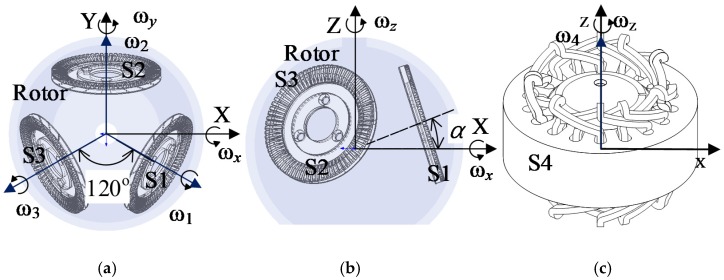
Motor structure: (**a**) Top view of piezoelectric stators/rotor structure, (**b**) Front view of piezoelectric stators/rotor structure, and (**c**) Structure of electromagnetic stator/rotor.

As shown in Figure 4a,b, the line passing through the No. 2 stator (S2) is defined as the Y-axis, the axis perpendicular to the Y-axis is the X-axis, and the line perpendicular to the X-Y plane and passing through the origin is defined as the Z-axis. The angle between the piezoelectric stators and the X-Y plane is α°.

As shown in Figure 4c, the electromagnetically-driven stator is mounted outside the spherical rotor. The rotor torque is generated by the interaction between the energized coil and the PM magnetic field. The 3-DOF motion is achieved by the synthesis of four stators motions. ω_x_, ω_y_, and ω_z_ are defined as the angular velocity vectors of the rotor rotating around the X, Y, and Z axes, respectively. According to the motor structure, the relation among **ω** [ω_x_, ω_y_, ω_z_] and the angular velocity vectors ω_1_, ω_2_, ω_3_, ω_4_ of the four stators can be expressed as:

(1) $$\omega =\left[\begin{array}{c} \omega_{x}\\ \omega_{y}\\ \omega_{z} \end{array}\right]=\left[\begin{array}{c} \frac{\sqrt{3}}{2}\omega_{1}\cos\alpha -\frac{\sqrt{3}}{2}\omega_{3}\cos\alpha \\ -\frac{1}{2}\omega_{1}\cos\alpha +\omega_{2}\cos\alpha -\frac{1}{2}\omega_{3}\cos\alpha \\ \omega_{1}\sin\alpha +\omega_{2}\sin\alpha +\omega_{3}\sin\alpha +\omega_{4} \end{array}\right]$$

## 3. Motor Design and Analysis

### 3.1. Modal Analysis

Modal analysis is an important issue for the motor dynamic properties. The modal analysis can predict and optimize the structural dynamics in product design. 

Through modal analysis, the pattern shape and the motor structure in the working frequency domain are identified. The basic structure of the motor is then determined.

The COMSOL finite element model of the motor is established in this paper. The structural parameters of the motor are as follows:

The piezoelectric stator is formed by bonding the elastic metal body and the piezoelectric ceramic. The number of teeth in the piezoelectric stator is 72. The stator materials are listed in Table 1. The elastic metal body (piezoelectric base body) material is phosphor bronze and the piezoelectric material is PZT-8.

The outer radius of the piezoelectric stators is 30 mm, the tooth height of the piezoelectric stators is 2 mm, the thickness of the piezoelectric ceramics is 0.5 mm and the thickness of the intermediate elastic body is 2.5 mm. The model of a piezoelectric stator is shown in Figure 3. Each tooth occupies a tooth height of 2 mm, a groove angle of 5° between the teeth, a circumferential angle of 3°, a tooth gap width of 5.5 mm, an elastic base thickness of 2.5 mm, and a piezoelectric ceramic thickness of 0.5 mm. The outer radius of the electromagnetic stator is 93.6 mm. The air gap between the electromagnetic stator and rotor is 1.5 mm. The inner radius of the rotor is 49 mm. The PM thickness is 10 mm. In order to predict the dynamic characteristics of the motor structure, the modal analysis of the stators and rotor is carried out. 

**Figure 5:**
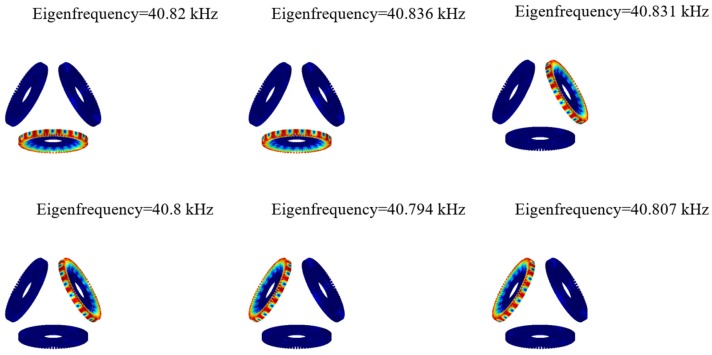
9th-order mode shape diagram of piezoelectric stator.

Based on the finite element model of the motor, the vibration frequency of the piezoelectric stator is obtained by modal analysis of a single stator. The specific steps are as follows: The solid mechanics module is used in the COMSOL finite element analysis software, and the material of the selected piezoelectric stator is shown in Table 1. The inner ring of the piezoelectric stator is set fixed, and the frequency analysis range is set to 20–60 kHz. The mode shape and frequency of the 9th order bending vibration mode of the piezoelectric stator are obtained through modal analysis. Figure 5 is a 9th-order modal shape diagram of a piezoelectric stator.

As shown in Figure 5, the modal frequency of the 9th-order mode of the motor is 40–41 kHz. When the motor rotor and the electromagnetic stator have a modal at 40–41 kHz, the motor rotor and the electromagnetic stator resonate, which causes the structural deformation of the rotor and reduces the performance of the motor. Therefore, in order to avoid the above problems, the solid mechanics module in the COMSOL finite element analysis software is used to perform modal analysis on the motor rotor and the electromagnetic stator. The specific steps are: the definition of the material parameters is shown in the table, and the boundary conditions are set to free boundary conditions. The frequency range is defined as 40–41 kHz. The outer diameter variable of the rotor is defined as wqR. The scanning parameter values of wqR are 51.5 mm, 52.5 mm, 53.5 mm, 54.5 mm, 55.5 mm.

**Figure 6:**
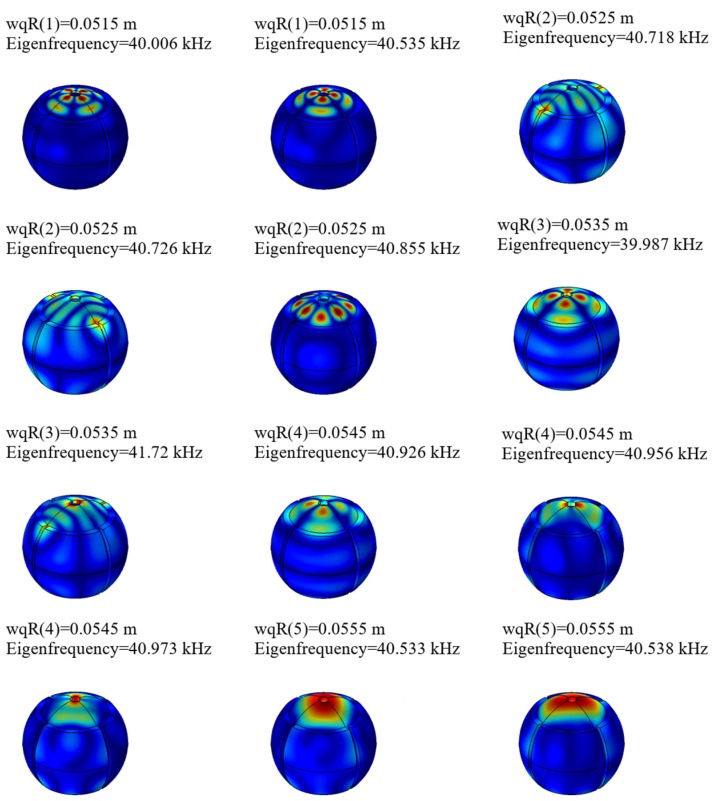
Rotor mode shape diagram.

As shown in Figure 6, when the outer radius is 53.5 mm, the rotor structure has two resonant modal modes with frequencies of 39.987 kHz and 41.72 kHz. The motor does not have any natural (Eigen) frequency between 40–41 kHz when the wqR is 53.5 mm. Then it can be concluded that the working frequency of the motor is not close to the resonant frequency.

**Figure 7:**
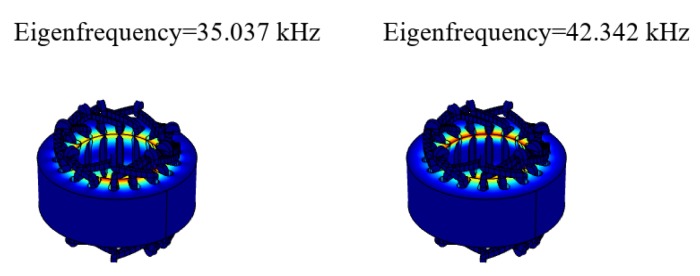
Electromagnetic stator mode shape diagram.

As shown in Figure 7, the electromagnetic stator has no vibration mode in the frequency range of 40–41 kHz. Therefore, the electromagnetic stator does not resonate, and the structure of the rotor will not be deformed.

### 3.2. Frequency Domain Analysis

The frequency domain analysis of the piezoelectric stator determines the steady-state response when it is excited by a sinusoidal voltage. The impedance and frequency variation and the electrical resonance frequency of the piezoelectric stator can also be obtained.

**Figure 8:**
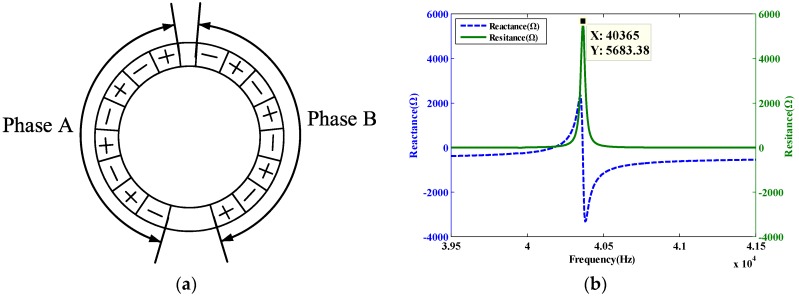
Motor frequency domain analysis chart: (**a**) layout of the applied load, (**b**) Motor frequency impedance diagram.

By using the solid mechanical analysis module in the COMSOL finite element analysis software, a voltage load is applied to the piezoelectric ceramics of the stator, and the form of the load is a plural form. The magnitude of the voltage on one side of the piezoelectric substrate is 0 V, and the magnitude of the voltage on the other side is 200 V. The layout of the applied load is shown in Figure 8a. The excitation voltage applied by phase A is 200 × exp(π/2 × i), and the excitation voltage applied by phase B is 200 × exp(0 × i). From the modal analysis results in Figure 5, the piezoelectric stator frequency is about at 40 kHz, so the frequency domain analysis is performed with 39.5–41.5 kHz. The frequency domain analysis result of the piezoelectric stator obtained by post-processing is shown in Figure 8.

As shown in Figure 8b, the piezoelectric stator reaches the resonance frequency at 40.365 kHz, and the frequency corresponding to the maximum operating speed of the motor is obtained.

### 3.3. Transient Analysis

The transient analysis of the piezoelectric stator utilizes a state equation that describes the continuous state change of the motor. The trajectory and vibration displacement of the piezoelectric stator are investigated by finite element analysis. Based on the frequency domain analysis, the phase A voltage excitation is defined as 200 × sin(2π × *f*), the phase B voltage excitation is defined as 200 × cos(2π × *f*), and *f* is 40.365 kHz. The material damping [24] is design by the damping ratio and frequency for modal analysis. The material parameters [25] of the piezoelectric ceramic sheet are: 

The ceramic piezoelectric constant matrix is

$$[e]=\left[\begin{array}{ccc} 0 & 0 & -3.875\\ 0 & 0 & -3.875\\ 0 & 0 & 13.91\\ 0 & 0 & 0\\ 0 & 10.344 & 0\\ 10.344 & 0 & 0 \end{array}\right]{\;C/m}^{2},$$

the dielectric constant matrix is

$$[\varepsilon^{s}\;]=\left[\begin{array}{ccc} 8.002 & 0 & 0\\ 0 & 8.002 & 0\\ 0 & 0 & 4.968 \end{array}\right]\times 10^{-9}F/m,$$

and the piezoelectric ceramic short-circuit stiffness matrix is

$$[c^{E}]=\left[\begin{array}{cccccc} 14.7 & 8.11 & 8.11 & 0 & 0 & 0\\ 8.11 & 13.2 & 7.3 & 0 & 0 & 0\\ 8.11 & 8.11 & 13.2 & 0 & 0 & 0\\ 0 & 0 & 0 & 3.29 & 0 & 0\\ 0 & 0 & 0 & 0 & 3.13 & 0\\ 0 & 0 & 0 & 0 & 0 & 3.13 \end{array}\right]\times 10^{10}\mathrm{Pa}.$$

**Figure 9:**
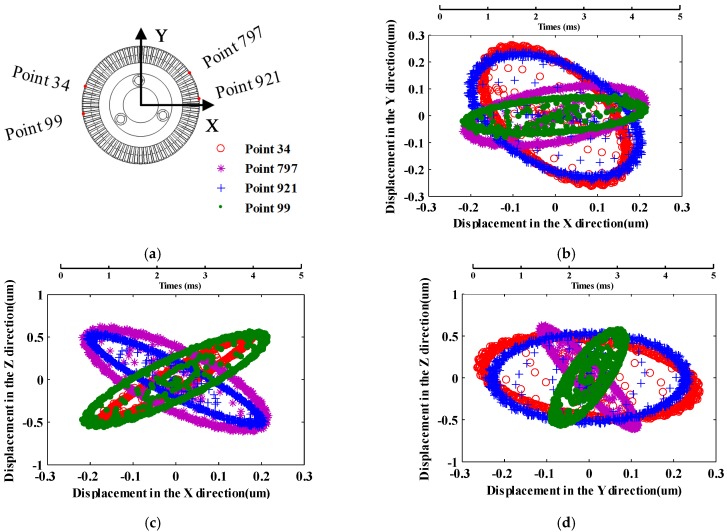
Trajectory diagram of the motor: (**a**) Schematic diagram of the point on the piezoelectric stator, (**b**) Trajectory on X-Y plane, (**c**) Trajectory on X-Z plane, and (**d**) Trajectory on Y-Z plane.

The movement of the motor stator at 0–5 ms is shown in the Figure 9. Four points in the piezoelectric stator drive boundary are selected, which is used to analyze the stator vibration characteristics, as shown in Figure 9a. It can be seen from Figure 9b–d that the trajectory of piezoelectric stator particle displacement is not elliptical at the beginning of the applied excitation. The displacement of the piezoelectric stator particle on the X-Z and Y-Z planes gradually increases, eventually reaching the maximum. Currently, the motion track of the particle becomes an elliptical. 

**Figure 10:**
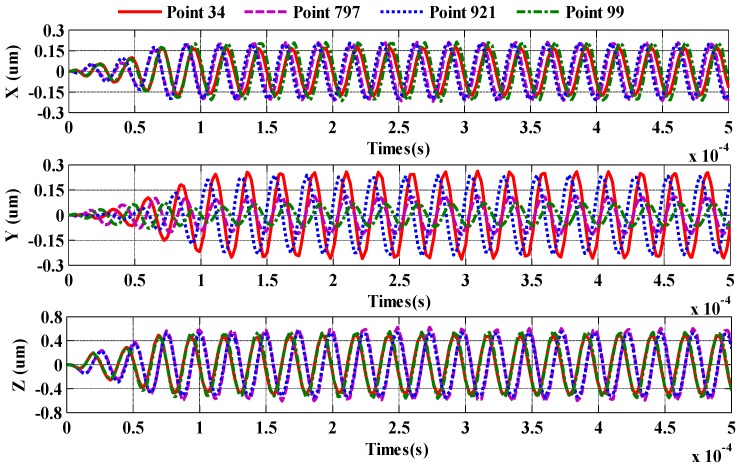
Piezoelectric stator time-displacement diagram.

It can be seen from Figure 10 that the displacement in the X, Y or Z direction is 0 µm at the time of t = 0. The displacement of the piezoelectric stator in the X, Y or Z direction reaches the maximum value within 0.1 ms, and the maximum value does not change. In other words, the time for the motor to start is 0.1 ms. The maximum displacement of the piezoelectric stator surface mass in the Z direction is about 0.5263 µm, and the results show that the displacement of the piezoelectric stator in the X and Y directions is about 0.1831 µm and 0.2443 µm, respectively. In order to generate a traveling wave that drives the rotor to rotate, the piezoelectric ceramic sheet is polarized along the thickness direction, that is, the Z direction, and the X and Y directions are not polarized. Therefore, when the piezoelectric ceramic sheet is excited, the Z-direction displacement is greater than the X- and Y-directions due to the inverse piezoelectric effect principle. The piezoelectric stator particle displacement in the X or Y direction is very small, so side shifting does not occur during the piezoelectric stator operation. It can be concluded that the piezoelectric stator structure is reasonable.

### 3.4. Piezoelectric Stator and Rotor Contact Analysis

**Figure 11:**
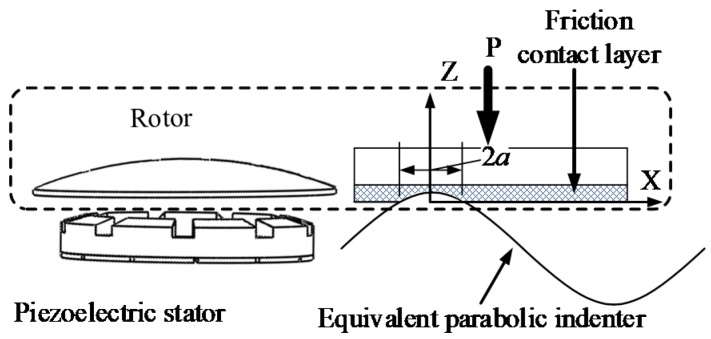
Piezoelectric Stator and rotor contact diagram.

In order to simplify calculation, the three-dimensional contact between the piezoelectric stator and the rotor is reduced to a one-dimensional problem (as shown in Figure 11). A wave contact between the piezoelectric stator and the rotor is analyzed.

The contact between the piezoelectric stator and the rotor is equivalent to the contact of the parabolic indenter with the friction contact layer, as shown in Figure 11. The piezoelectric stator friction contact layer is set to be displaced in the Z-axis direction to the maximum displacement when the piezoelectric stator is in contact with the rotor. The piezoelectric stator is a rigid body and does not displace, so the friction between the piezoelectric stator and rotor is ignored.

The waveform of the piezoelectric stator at a certain moment is:

(2) $$u_{1}=\mathrm{Acos}\left(kx-\omega t\right)$$

The curvature radius *R_c_* of this waveform is [26]:

(3) $${{}_{R}}_{c}=\left|{\left[1+{\left(\frac{du_{1}}{dx}\right)}^{2}\right]}^{\frac{3}{2}}/\frac{d^{2}u_{1}}{dx^{2}}\right|$$

The pre-pressure applied by each stator is F, and then the one-dimensional pre-stress experienced by each peak is the fixed-rotor contact zone displacement function, which can be defined as:

(4) $$w=-\frac{x^{2}}{2R_{c}}+d_{0}\;,z=0,\left|x\right|\leq a$$

where, *d*_0_ is an unknown constant and, *a* is half the contact distance of the fixed rotor within one wavelength of the piezoelectric stator.

The friction between the piezoelectric stator and the rotor is neglected, and the shear force at the piezoelectric stator and rotor boundary is zero. That is x = 0, *τ*_xy_ = 0.

The piezoelectric stator and rotor elastic contact normal stress distribution is *p*(x), z = 0, |x| ≤ a. 

According to the balance of the piezoelectric stator and rotor:

(5) $$\int_{-a}^{a}p\left(x\right)dx=\frac{F}{nb}$$

where, *n* is the number of traveling waves, and *b* is the equivalent width of the piezoelectric stator contact.

When the fixed rotor contact force *p(ξ)* acts, the vertical displacement expression [26] is:

(6) $$\frac{-1}{\pi E}\int_{-a}^{a}\ln{\left(x-\xi \right)}^{2}p\left(\xi \right)d\xi =-\frac{x^{2}}{2R_{c}}+d_{0}$$

where, E is the Young’s modulus of the friction layer.

By solving the first type of Fredholm equation and solving the boundary conditions [27,28,29], the rotor contact force distribution is as follows:

(7) $$p\left(x\right)={\left(\frac{PE}{\pi R_{c}a^{2}}\right)}^{\frac{1}{2}}{\left(a^{{}_{2}}-x^{2}\right)}^{\frac{1}{2}}$$

The piezoelectric stator-rotor contact surface displacement is:

(8) $$u=-\frac{x^{2}}{2R_{c}}+\frac{P}{\pi E}\left(2\ln2+1\right)$$

The parameters of the piezoelectric stator are as shown in Table 2. 

**Figure 12:**
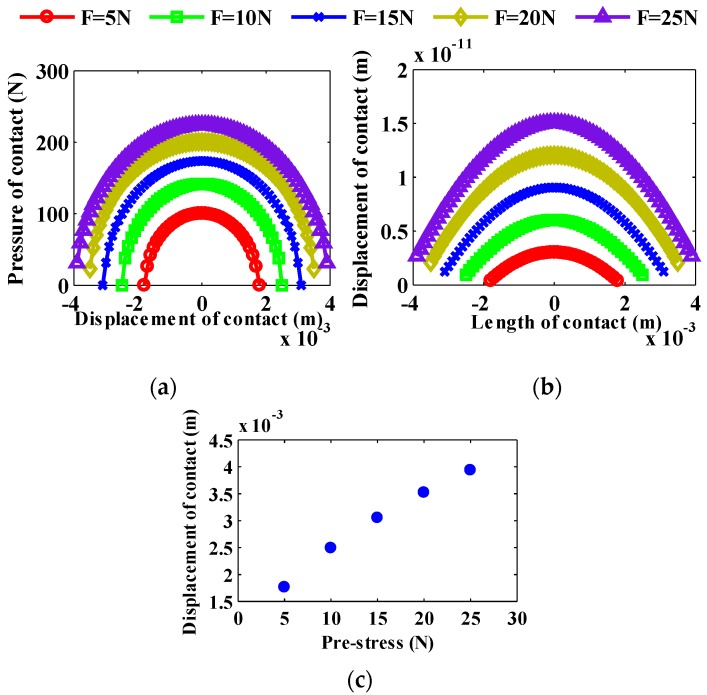
Piezoelectric stator and rotor contact: (**a**) Pressure vs. displacement of the contact under different pre-pressures, (**b**) Displacement of contact surface vs. contact length under different pre-pressures, and (**c**) Displacement of contact vs. pre-pressure.

According to the data in Table 2, from Equations (7) and (8), we use MATLAB software to numerically simulate the contact pressure and contact surface distribution of the stator and rotor of the motor. The piezoelectric stator-rotor contact displacements calculated with the parameters are shown in Figure 12a–c. It can be seen from Figure 12a–c that the greater the pre-pressure, the greater the length of the rotor contact. The greater the pre-pressure, the greater the displacement of the pressure contact point. The greater the pre-pressure, the greater the pressure at the contact point.

### 3.5. Modeling and Analysis of Rotor Air Gap Magnetic Field 

**Figure 13:**
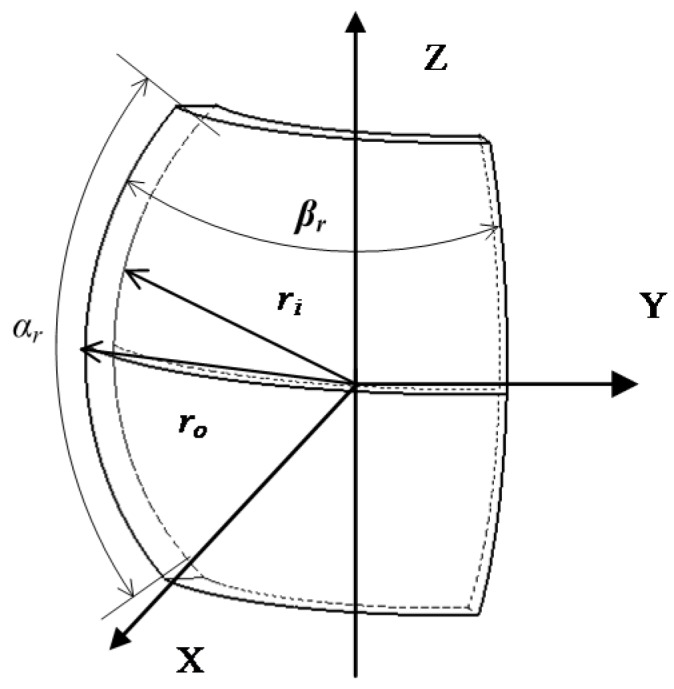
Permanent magnet structure diagram of the rotor.

The rotor PM structure is shown in Figure 13. According to the magnetic medium boundary conditions, the magnetic field generated by the rotor permanent magnet can be divided into two parts:

(9) $$\{\begin{array}{cc} B_{1}=\mu_{0}\mu_{r}H_{1} & r\geq R_{o}\\ B_{2}=\mu_{0}\mu_{m}H_{2}+\mu_{0}M_{r} & R_{i}\leq r<R_{o} \end{array}$$

where, *μ*_0_ is the magnetic permeability in vacuum, its value is 4π × 10^−7^ H/m, *μ*_m_ is the relative magnetic permeability of the permanent magnet, and its value is close to 1, and *μ*_r_ is the relative permeability of air, its value is also close to 1.

The PM material is NdFeB35, **M_r_** is the residual magnetization. |**M**_r_| = *B*_r_/*μ*_0_ (A/m), *B*_r_ is the residual magnetic flux density with a value of 1.17 T.

The parallel permanent magnet magnetization vector is:

(10) $$M_{r}=\{\begin{array}{c} M_{rr}\\ M_{r\theta }\\ M_{r\phi } \end{array}\}={\left(-1\right)}^{p-1}\left|M_{r}\right|\{\begin{array}{c} \cos\left(\varphi -\alpha_{p}\right)\sin\theta \\ \cos\left(\varphi -\alpha_{p}\right)\cos\theta \\ -\sin\left(\varphi -\alpha_{p}\right) \end{array}\}$$

(11) $$\alpha_{p}=\alpha_{r}/2+2\pi (p-1)/L$$

(12) $$\pi /2-\beta_{r}/2\leq \theta \leq \pi /2+\beta_{r}/2$$

(13) $$\pi \left(p-1\right)/L\leq \varphi \leq \alpha_{r}+\pi \left(p-1\right)/L$$

where *p* is the permanent magnet number (*p* = 1, 2, 3, 4), *L* is the total number of permanent magnets, *α_r_* is the permanent magnet with a longitude angle of 41° and *β_r_* is the permanent magnet with a latitude angle of 85°.

The PM mounting positions are symmetrically distributed, hence

(14) $$\nabla \cdot M_{r}=0$$

There is no current excitation in the rotor area, and we have:

(15) $$\nabla \times H_{i}=0$$

(16) $$H_{i}=-\nabla \Phi_{i}$$

where, *i* = 1, 2.

The magnetic scalar potentials in the two regions can be expressed as:

(17) $$\nabla^{2}\Phi_{1}=0$$

(18) $$\nabla^{2}\Phi_{2}=0$$

The Laplace equation of scalar magnetic potentials Φ*_i_* can be expressed as:

(19) $$\frac{1}{r^{2}}\frac{\partial }{\partial r}\left(r^{2}\frac{\partial \Phi_{i}}{\partial r}\right)+\frac{1}{r^{2}\sin\theta }\frac{\partial }{\partial \theta }\left(\sin\theta \frac{\partial \Phi_{i}}{\partial \theta }\right)+\frac{1}{r^{2}\sin^{2}\theta }\frac{\partial^{2}\Phi_{i}}{\partial \varphi^{2}}=0$$

The solution of the magnetic potential equation by using the variable separation method is obtained as:

(20) $$\begin{array}{l} \Phi_{i}(r,\theta ,\varphi )=R(r)Y(\theta ,\varphi )\\ =\sum_{n=0}^{+\infty }\sum_{m=0}^{n}\left(C_{n}r^{n}+\frac{D_{n}}{r^{n+1}}\right)P_{n}^{m}(\cos\theta )(A_{m}\cos m\varphi +B_{m}\sin m\varphi )\\ =\sum_{n=0}^{+\infty }\sum_{m=-n}^{n}\left(C_{n}r^{n}+\frac{D_{n}}{r^{n+1}}\right)Y_{n}^{m}\left(\theta ,\varphi \right) \end{array}$$

where, *i* = 1,2. $Y_{n}^{m}\left(\theta ,\varphi \right)=S_{n}^{m}P_{n}^{m}\left(\cos\theta \right)e^{im\varphi }$ (*m* = 0, 1, … *n*), $S_{n}^{m}=\sqrt{\frac{2n+1}{4\pi }\frac{\left(n-m\right)!}{\left(n-m\right)!}}$, $Y_{n}^{m}\left(\theta ,\varphi \right)$ is a spherical harmonic function, $P_{n}^{m}\left(\cos\theta \right)$ is the associated Legendre function.

The boundary conditions for the solution are:

(21) $$\Phi_{1}|{}_{r\to \infty }=0$$

(22) $$B_{1r}=B_{2r}|r=r_{o}$$

(23) $$H_{1\theta }=H_{2\theta }|r=r_{o}$$

(24) $$H_{1\phi }=H_{2\phi }|r=r_{o}$$

The magnetic field strength in spherical coordinates is:

(25) $$H_{i}={\left[H_{r}e_{r},H_{\theta }e_{\theta },H_{\varphi }e_{\varphi }\right]}^{T}={\left[-\frac{\partial \Phi_{i}}{\partial r},-\frac{1}{r}\frac{\partial \Phi_{i}}{\partial \theta },-\frac{1}{r\sin\theta }\frac{\partial \Phi_{i}}{\partial \varphi }\right]}^{T}$$

where, *i* = 1,2.

The fundamental component of flux density can be obtained by solving the boundary conditions as

(26) $$B_{11}=\left[\begin{array}{c} B_{11r}\\ B_{11\theta }\\ B_{11\varphi } \end{array}\right]=\mu_{0}\mu_{r}r^{-4}D_{n,1}^{m}S_{n}^{m}\left[\begin{array}{c} 3{\left(\sin\theta \right)}^{2}\sin2\varphi \\ -2\sin\theta \cos\theta \sin2\varphi \\ -2\sin\theta \cos2\varphi \end{array}\right]$$

**Figure 14:**
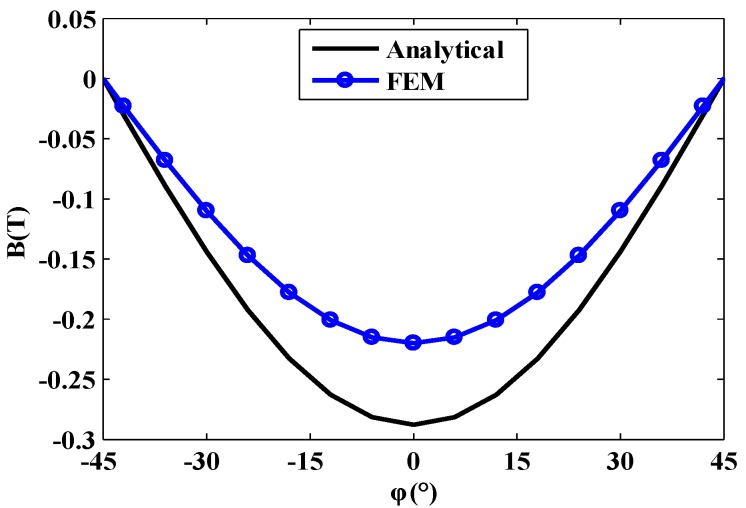
Finite element and analytical comparison analysis of air gap magnetic field.

In order to verify the accuracy of the magnetic field analysis, the air gap magnetic field at the distance of 0.5 mm from the permanent magnet is taken, and the comparative analysis is carried out by the finite element method (FEM). The results are shown in Figure 14. The results of the analytical method and the FEM are in relative agreement. The feasibility of the analytical method is verified, which provides a basis for the fast analysis and optimization of the motor prototype.

## 4. Driver Model of Electromagnetic-Piezoelectric-Hybrid Drive Motor

The mathematical driving model of the electromagnetic-piezoelectric-hybrid drive three-degree-of-freedom motor is composed of two parts: the electromagnetic drive and the piezoelectric drive.

### 4.1. Piezoelectric Drive Modeling

According to the Mindlin theory [30,31,32,33], the tangential force distribution of the stator and rotor is:

When T_max_ ≤ $\mu \left(\frac{\mathrm{PE}}{\pi R}\right)$, the tangential force distribution is provided by the following equation:

(27) $$\{\begin{array}{l} f\left(x\right)=-\mu {\left(\frac{\mathrm{PE}}{\pi R}\right)}^{\frac{1}{2}}{\left(1-\frac{x^{2}}{a^{2}}\right)}^{\frac{1}{2}}c\leq \left|x\right|\leq a\\ f\left(x\right)=-\mu {\left(\frac{\mathrm{PE}}{\pi R}\right)}^{\frac{1}{2}}\left[{\left(1-\frac{x^{2}}{a^{2}}\right)}^{\frac{1}{2}}-2\frac{c}{a}\left(1-\frac{x^{2}}{c^{2}}\right)\right]c_{\max}\leq \left|x\right|\leq a\\ f\left(x\right)=-\mu {\left(\frac{\mathrm{PE}}{\pi R}\right)}^{\frac{1}{2}}\left[{\left(1-\frac{x^{2}}{a^{2}}\right)}^{\frac{1}{2}}-2\frac{c}{a}\left(1-\frac{x^{2}}{c^{2}}\right)+\frac{c_{\max}}{a}\left(1-\frac{x^{2}}{c_{\max}{}^{2}}\right)\right]\left|x\right|\leq c_{\max} \end{array}$$

where *c*_max_ is $\frac{c_{\max}}{a}={\left(1-\frac{T_{\max}}{\mu \frac{\mathrm{PE}}{\pi R}}\right)}^{\frac{1}{2}}$, $\frac{c}{a}={\left(1-\frac{T_{\max}-T}{\mu \frac{\mathrm{PE}}{\pi R}}\right)}^{\frac{1}{2}}$, *μ* is the dynamic friction, *T* is the resistance of the motor movement, *T*_max_ is the maximum resistance of the motor operation and *c* is a function about *T*.

When *T*_max_ = $\mu \left(\frac{\mathrm{PE}}{\pi R}\right)$, the tangential force distribution is provided by the following equation:

(28) $$\{\begin{array}{l} f\left(x\right)=-\mu {\left(\frac{\mathrm{PE}}{\pi R}\right)}^{\frac{1}{2}}{\left(1-\frac{x^{2}}{a^{2}}\right)}^{\frac{1}{2}}c\leq \left|x\right|\leq a\\ f\left(x\right)=-\mu {\left(\frac{\mathrm{PE}}{\pi R}\right)}^{\frac{1}{2}}\left[{\left(1-\frac{x^{2}}{a^{2}}\right)}^{\frac{1}{2}}-2\frac{c}{a}\left(1-\frac{x^{2}}{c^{2}}\right)\right]c_{\max}\leq \left|x\right|\leq a \end{array}$$

where, $\frac{c}{a}={\left(1-\frac{T_{\max}-T}{\mu \frac{\mathrm{PE}}{\pi R}}\right)}^{\frac{1}{2}}$.

Under the normal contact pressure in the contact area, the tangential thrust *F_t_* on the resultant rotor is:

(29) $$F_{t}=\int_{-a}^{a}f(x)dx$$

The output torque is

(30) $$T_{j}=nRF_{t}$$

where *n* is the number of traveling wave peaks, *R* is the outer radius of the piezoelectric stators, and *j* (*j* = 1, 2, 3) is the number of the piezoelectric stators.

### 4.2. Electromagnetic Drive Modeling

The magnetic field generated by the energized coil interacts with the magnetic field generated by the PMs, producing an interaction Lorentz force. The electromagnetic force generated by a coil *dl* micro-element can be obtained by the Lorentz force law. Then, the electromagnetic torque generated by the *dl* micro-element is calculated, and the torque micro-element is integrated to obtain the torque generated by the single coil. Finally, the rotation torque of the electromagnetic stator drive is obtained [29]:

(31) $$T_{4}=\sum_{i=1}^{N}J_{k}\int_{r_{1}}^{r_{2}}\int_{\delta_{0}}^{\delta_{1}}\int_{0}^{2\pi }B_{11r}(r,\theta_{i},\varphi_{i},\phi ,\delta )\sin\delta \sin\phi d\phi drd\theta$$

where *k* is the coil number, and *N* is the total number of coils.

### 4.3. Electromagnetic-Piezoelectric Hybrid Motor Modeling

As shown in Figure 4, the line passing through the No. 2 stator (S2) is defined as the Y-axis, the axis perpendicular to the Y-axis is the X-axis, and the line perpendicular to the X-Y plane and passing through the origin is defined as the Z axis, and the angle between the piezoelectric stators and the X-Y plane is α°, the torque of the three-degree-of-freedom motor is **T**, which is synthesized by the torque vectors *T*_1_, *T*_2_, *T*_3_ and *T*_4_ of the four stators:

(32) $$T=\left[\begin{array}{c} T_{x}\\ T_{y}\\ T_{z} \end{array}\right]=\left[\begin{array}{c} \frac{\sqrt{3}}{2}T_{1}\cos\alpha -\frac{\sqrt{3}}{2}T_{3}\cos\alpha \\ -\frac{1}{2}T_{1}\cos\alpha +T_{2}\cos\alpha -\frac{1}{2}T_{3}\cos\alpha \\ T_{1}\sin\alpha +T_{2}\sin\alpha +T_{3}\sin\alpha +T_{4} \end{array}\right]$$

## 5. Experimental Verification and Analysis

**Figure 15:**
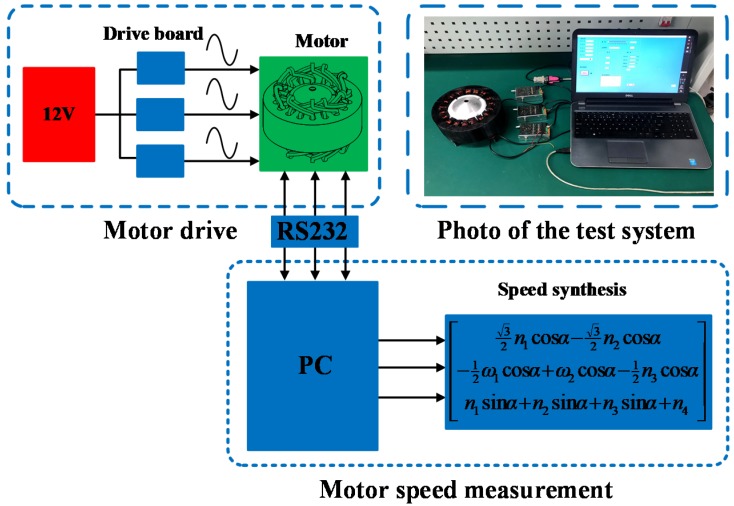
Prototype experimental test system.

In order to verify the rationality of the motor structure and performance analysis, a prototype experimental test system is built as shown in Figure 15. The system includes a 3-DOF prototype of an electromagnetic-piezoelectric-hybrid drive motor, a driver and a host computer. The driver used in this experiment is UMD-3II. The power line of the DC12V power supply is connected to the UMD-3II driver power port, the A phase of the ceramic chip is connected to the sine port of the driver, and the cosine port of the B phase driver of the ceramic chip. The UMD-3II driver uses RS232 serial communication. The serial communication line of the driver is connected to the host computer. Adjust the frequency of the excitation voltage to measure the speed of the motor on the host computer. The rotation speed measured by this platform is the rotation speed (*n*_1_, *n*_2_, *n*_3_) around the central axis of the stator. *n*_x_, *n*_y_, can be calculated by Equation (33).

(33) $$n=\left[\begin{array}{c} n_{x}\\ n_{y}\\ n_{z} \end{array}\right]=\left[\begin{array}{c} \frac{\sqrt{3}}{2}n_{1}\cos\alpha -\frac{\sqrt{3}}{2}n_{3}\cos\alpha \\ -\frac{1}{2}n_{1}\cos\alpha +n_{2}\cos\alpha -\frac{1}{2}n_{3}\cos\alpha \\ n_{1}\sin\alpha +n_{2}\sin\alpha +n_{3}\sin\alpha +n_{4} \end{array}\right]$$

**Figure 16:**
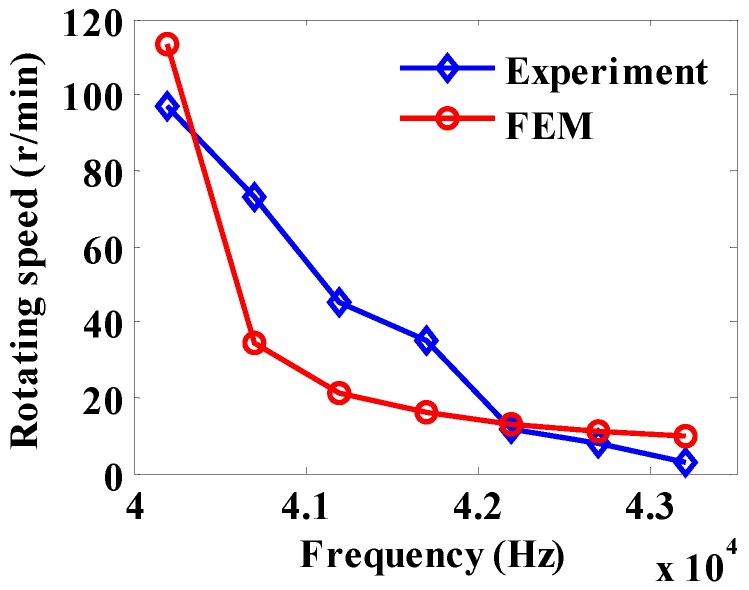
Speed vs. frequency diagram of piezoelectric stator.

Figure 16 is a graph of the rotation speed *n*_1_ with frequency. The FEM rotation speed *n*_1_ is obtained according to a previous method [34]. The experimental and FEM methods are in good agreement. It can be seen from Figure 16 that the rotation speed of the motor decreases as the frequency increases.

**Figure 17:**
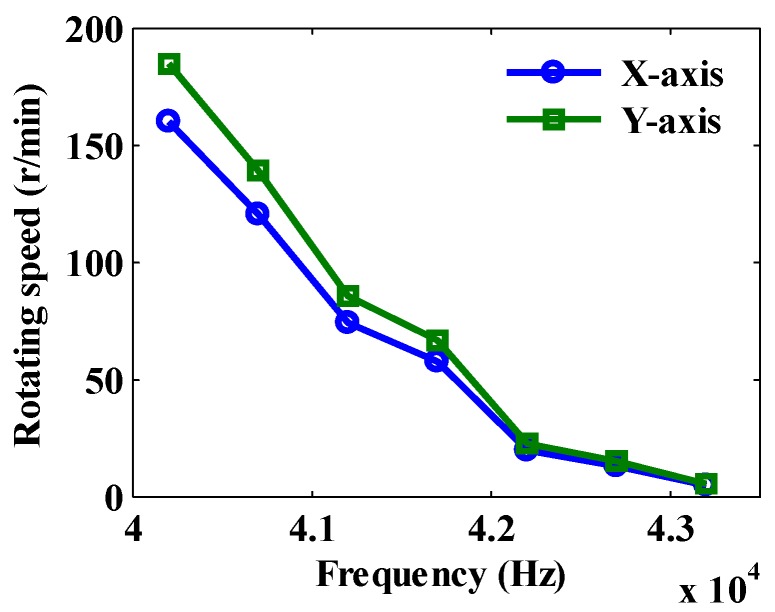
Synthetic speed vs. frequency diagram.

According to Equation (33), the electromagnetic waves of No. 1 and No. 3 stators (S1 and S3) are driven in the forward direction, and that of No. 2 stator (S2) is reversely driven to obtain the rotation speed of the motor at different voltage excitation frequencies, as shown in Figure 17. As can be seen from Figure 17, the maximum speed of motor is about 40.2 kHz, which is the approximate resonant frequency of a piezoelectric stator. It can be known from the experimental results that the speed of the motor decreases as the frequency increases, so the speed can be adjusted by changing the voltage excitation frequency.

## 6. Conclusions

A novel electromagnetic-piezoelectric-hybrid drive three-degree-of-freedom motor was proposed. The structural model was established by finite element analysis software, and the modal analysis, harmonic response analysis and transient analysis of the motor were conducted. The magnetic field characteristics are analyzed and compared by analytical and finite element methods. The contact pressure and contact displacement between the piezoelectric stator and the rotor were analyzed by the analytical method, and the motor drive model was established. The rationality and feasibility of the design are verified by the experimental results on a prototype. The technical scheme of the electromagnetic-piezoelectric-hybrid three-degree-of-freedom motor is feasible, and it has research value. In the next step, it is planned to investigate further the electromagnetic-piezoelectric-hybrid drive mechanism for more effective modeling and system-level multi-discipline optimization for high-performance three-DOF motor drives.

## Figures and Tables

**Table 1:** Main Parameters of Stators and Rotor Materials.

Motor Component	Material	Young’s Modulus (N/m^2^)	Poisson’s Ratio	Density (kg/m^3^)
Piezoelectric ceramics	PZT-8	112 × 10^9^	0.33	7600
Piezoelectric base body	Phosphor bronze	8 × 10^9^	0.3	8800
Rotor material	Hard aluminum	72 × 10^9^	0.33	2700
Rotor PM	NdFeB35	16 × 10^10^	0.3	7500
Electromagnetic stator coil	Copper	11 × 10^10^	0.35	8960
Electromagnetic stator core	Silicon steel	2 × 10^11^	0.25	7600

**Table 2:** Structural Parameters of piezoelectric stator.

Parameters	Value (units)
*b*	2 mm
*λ*	6.67 π
*f*	40.365 kHz
*V*	200 V
*n*	9
*E*	72 × 10^9^ N/m^2^
